# Chemical and elemental mapping of spent nuclear fuel sections by soft X-ray spectromicroscopy

**DOI:** 10.1107/S1600577521012315

**Published:** 2022-01-01

**Authors:** Alexander Scott Ditter, Danil E. Smiles, Daniel Lussier, Alison B. Altman, Mukesh Bachhav, Lingfeng He, Michael W. Mara, Claude Degueldre, Stefan G. Minasian, David K. Shuh

**Affiliations:** a Chemical Sciences Division, Lawrence Berkeley National Laboratory, One Cyclotron Road, Berkeley, CA 94720, USA; b Department of Chemistry, University of California Berkeley, 420 Lattimer Hall, Berkeley, CA 94720, USA; c Idaho National Laboratory, 1955 N. Freemont Avenue, Idaho Falls, ID 83415, USA; d Department of Engineering, Lancaster University, Lancaster, Lancashire LA1 4YW, United Kingdom

**Keywords:** spent nuclear fuel, STXM, oxygen *K*-edge, focused ion beam sections

## Abstract

Soft X-ray spectromicroscopy at the O *K*-edge, U *N*_4,5_-edges and Ce *M*_4,5_-edges has been performed on focused ion beam sections of spent nuclear fuel for the first time, yielding chemical information on the sub-micrometer scale. To analyze these data, a modification to non-negative matrix factorization (NMF) was developed, in which the data are no longer required to be non-negative, but the non-negativity of the spectral components and fit coefficients is largely preserved. The modified NMF method was utilized at the O *K*-edge to distinguish between two components, one present in the bulk of the sample similar to UO_2_ and one present at the interface of the sample which is a hyperstoichiometric UO_2+*x*_ species. The species maps are consistent with a model of a thin layer of UO_2+*x*_ over the entire sample, which is likely explained by oxidation after focused ion beam (FIB) sectioning. In addition to the uranium oxide bulk of the sample, Ce measurements were also performed to investigate the oxidation state of that fission product, which is the subject of considerable interest. Analysis of the Ce spectra shows that Ce is in a predominantly trivalent state, with a possible contribution from tetravalent Ce. Atom probe analysis was performed to provide confirmation of the presence and localization of Ce in the spent fuel.

## Abstract 2

Soft X-ray spectromicroscopy at the O *K*-edge, U *N*_4,5_-edges and Ce *M*_4,5_-edges has been performed on focused ion beam sections of spent nuclear fuel for the first time. Analysis of oxygen spectra using a modified non-negative matrix factorization method is consistent with a thin layer of hyperstoichiometric uranium oxide having been formed at the interface of a sample consisting of primarily UO_2_, probably formed after sample preparation. The cerium oxidation state is shown to be predominantly trivalent, with a possible small contribution from tetravalent Ce.

## 1. Introduction

Spent nuclear fuel is a complex environment of uranium, plutonium, minor actinides and a host of fission products, combined with stresses and large thermal gradients (Kleykamp, 1985 ▸; Olander, 1976 ▸; Bruno & Ewing, 2006 ▸; Janeczek *et al.*, 1996 ▸). Understanding this chemical environment is key to developing advanced nuclear fuels for safer and higher-performing reactors. Uranium oxidation in particular is important as the fission process causes an excess of oxygen to build up in the fuel, and this partial pressure of oxygen is important in understanding fuel-cell cladding chemical interaction and the thermal conductivity of the fuel (Lucuta *et al.*, 1995 ▸; Walker *et al.*, 2005 ▸; Olander, 1976 ▸). Because *in situ* measurements in a nuclear reactor are not possible, studying spent nuclear fuel after removal is one of the best ways to gain insight into this complicated environment. Oxidation of the spent fuel itself is also important, as this affects the release of fission gases, swelling of the fuel, and the dissolution of uranium and the fission products under environmental conditions (Ewing, 2015 ▸; Hastings *et al.*, 1986 ▸; Tonks *et al.*, 2018 ▸).

The fission products are another particularly interesting aspect of the nuclear fuel. These are broadly categorized into fission gases like Xe and Kr, noble metal precipitates like Rh and Tc, solid-state precipitates like Ba and Zr, and solid-solution products like Ce and Pm (Kleykamp, 1985 ▸). Ce is particularly interesting due to its potential redox interactions with the uranium oxide matrix (Markin *et al.*, 1970 ▸; Hanken *et al.*, 2011 ▸). There is ongoing debate over whether uranium oxide can reduce Ce in a spent fuel environment, with several idealized U_*x*_Ce_1–*x*_O_*y*_ systems studied with X-ray photo­electron spectroscopy, X-ray absorption spectroscopy and density functional theory (DFT) (Griffiths *et al.*, 1994 ▸; Eloirdi *et al.*, 2018 ▸; Antonio *et al.*, 1996 ▸; Tracy *et al.*, 2015 ▸; Suresh Kumar *et al.*, 2004 ▸; Bera *et al.*, 2009 ▸). These studies show that reduction of Ce in a UO_2_ matrix is possible, but this depends on factors such as temperature, the exact redox potential in the sample’s environment, and the relative concentrations of Ce and U. To the best of our knowledge, prior to this work there have been no direct measurements of the Ce oxidation state in spent fuel.

Soft X-ray spectromicroscopy using a scanning transmission X-ray microscope (STXM) combines the chemical sensitivity of soft X-ray spectroscopy with the spatial resolution necessary to resolve features of the order of tens of nanometres (Hitchcock, 2015 ▸; Sakdinawat & Attwood, 2010 ▸; Jacobsen *et al.*, 2000 ▸, 1991 ▸). Previous soft X-ray spectroscopy studies on uranium oxides have shown a diversity of spectral features, making the oxygen *K*-edge suitable for fingerprinting uranium oxidation states (Conradson *et al.*, 2013 ▸; Frati *et al.*, 2020 ▸). Cerium similarly shows a clear difference at the *M*_4,5_-edge between trivalent and tetravalent oxidation states (Smiles *et al.*, 2020 ▸; Kaindl *et al.*, 1984 ▸). The main experimental hurdle in soft X-ray STXM is that the optimal thickness for STXM measurements of uranium oxide at the oxygen *K*-edge and U *N*-edges is a few hundred nanometres (Gullikson, 2010 ▸).

Focused ion beam (FIB) sectioning is one way to prepare STXM samples in a way that preserves the structure of the sample yet allows for optimal thickness for soft X-ray spectroscopy at 100–200 nm. Although the FIB sections produced are relatively uniform in thickness, defects like voids and cracks present in the sample before FIB sectioning offer interesting points for investigation and useful references for image alignment, which is crucial for STXM analysis. FIB sections have been studied on a variety of samples from environmental (Benzerara *et al.*, 2007 ▸) to archeological (Michelin *et al.*, 2013 ▸; Bernard *et al.*, 2009 ▸, 2007 ▸) to interplanetary (Yabuta *et al.*, 2014 ▸; Uesugi *et al.*, 2014 ▸; Ito *et al.*, 2020 ▸; Gu *et al.*, 2020 ▸). FIB sections have also been used to study spent nuclear fuel recently (Clark *et al.*, 2020 ▸; Kessler *et al.*, 2020 ▸; Yuan *et al.*, 2021 ▸; Liu *et al.*, 2021 ▸; Teague & Gorman, 2014 ▸; Degueldre *et al.*, 2016 ▸; Degueldre & Veleva, 2014 ▸). These studies have mainly utilized transmission electron microscopy (TEM) and transmission extended X-ray absorption fine structure (EXAFS), which also require thin samples, especially TEM. When working with nuclear fuel, preparing FIB sections has the additional benefit of only yielding a small amount of material which limits the overall radioactivity of the sample, making studies safer and limiting containment efforts. Put together, STXM spectromicroscopy of FIB sections is an ideal method for determining chemical information in spent nuclear fuel on the nanoscale. The measurements described here represent the first soft X-ray STXM measurements of spent nuclear fuel.

## 2. Methods

### 2.1. Sample preparation

The FIB sections were prepared from both the center and rim areas of a spent light-water reactor fuel from Belgium Reactor 3 (BR3) with an average burnup of 4.5 at%, translating to approximately 40 MW d kg^−1^ (Herrmann *et al.*, 2007 ▸). Cross sections were prepared in a Quanta 3D FEG FIB system by the following procedure. A protective platinum layer was first deposited onto the surface. FIB sections were then created by coarse trenching a 15 µm × 10 µm × 1 µm sample using the FIB. The section was then welded to a copper TEM grid and thinned by 30 keV Ga ions with progressively decreasing current. Two TEM grids were generated, one from the rim of the pellet with two lamellae attached to it (1A and 1B) and one from the center of the pellet with two lamellae attached to it (2A and 2B). The FIB sections were thinned to ∼100 nm thick and the final thinning current was ∼100 pA. The FIB sections were then cleaned with 5 keV Ga ions and finally with 2 keV Ga ions. In one FIB section (1B), the ion beam was used to produce a hole in the section.

Preliminary microstructure was characterized using the Titan Themis 200 TEM before the STXM study. Specimens for atom-probe tomography (APT) were prepared using standard lift-out and milling procedures using an FEI Quanta 3D FEG scanning electron microscope and FIB instrument located in the Electron Microscopy Laboratory (EML) at Idaho National Laboratory (INL) (Thompson *et al.*, 2007 ▸).

The thin FIB section samples on the Cu grids were transferred to Lawrence Berkeley National Laboratory, where they were stored under ambient atmosphere for ten days. During this time or during shipment, these samples were exposed to air and therefore probably oxidized on the surface, as is typical for uranium oxide. The Cu grids were affixed to an aluminium sample plate using five-minute epoxy. The Advanced Light Source (ALS) has protocols in place ensuring the safe handling of radioactive materials, including restrictions on sample activity and containment requirements. Because of the small size of the samples, the total activity for all four sections was reduced to 3 × 10^−10^ Ci, which meant that additional containment of the sample was not required.

### 2.2. X-ray absorption spectroscopy data collection

Data were collected on ALS Beamline 11.0.2 using the STXM of the Molecular Environmental Sciences endstation (Bluhm *et al.*, 2006 ▸). This endstation operates between 100 and 2000 eV, with a spectral resolution *E*/Δ*E* > 10000. Data were collected with the synchrotron operating under normal user conditions (1.9 GeV, 500 mA, top-off mode, multi-bunch). Samples were loaded into the STXM, and images of the sample were collected in transmission by rastering the sample through the beam. Stacks of images at many different energies were assembled at different locations on each of the four FIB sections. Oxygen stacks were collected between 500 and 600 eV with an energy spacing of 0.15 eV from 528 eV to 541 eV. Uranium spectra were taken between 711 and 806 eV with an energy spacing of 0.5 eV from 727 to 748 eV and 772 to 787 eV. Cerium spectra were collected between 870 and 920 eV with an energy spacing of 0.2 eV from 878 to 905 eV. Data were calibrated in energy to a Rydberg feature of the O *K*-edge spectrum of CO_2_ at 539.8 eV (Prince *et al.*, 1999 ▸). Data were collected at room temperature.

### 2.3. Data analysis

During data collection, the location of the focus of the zone plate changes slightly as the energy is changed. Thus, aligning the images is necessary. The alignment of images for each stack was handled using *aXis2000* (http://unicorn.mcmaster.ca/aXis2000.html). This was only possible for stacks where the FIB section had a hole or other identifying features. For other stacks, alignment was not possible, and data collected in those regions were deemed too uncertain to process reliably. The drift of the zone plate for different stacks took the same general path but was not consistent enough to apply to stacks without identifying features. One exception is the O *K*-edge stack taken on FIB section 1A, which is centered on an intergranular crack. Although the crack itself is too small to resolve with STXM, the difference in the oxygen spectra between UO_2_ and hyperstoichiometric uranium oxide allows for manual image alignment, using areas near the crack, but the automatic routines of *aXis2000* were not able to detect these differences.

Once the images were aligned, the data were converted to optical density using the incident flux *I*_0_. Where possible, this was done by selecting a region in the stack with no sample, either beyond the edge of the sample or in a hole in the sample. When there was no such region, a separate scan completely outside the FIB section was used. Normalizing this way is less ideal because short-term fluctuations in the incident flux are not accounted for. Averages over large areas of the sample (*e.g.*, Ce *M*_4,5_ spectra or U *N*_4,5_ spectra) were done using *aXis2000*. Care was taken to select areas of similar thickness to avoid pinhole effects (Achkar *et al.*, 2011 ▸). Pinhole effects occur when averaging over a region of the sample which contains some thick regions and some thin regions. The non-linearity of Beer’s law results in a suppression of peak intensity on samples with pinholes. In some regions (O *K*-edge FIB section 1B, U *N*_4,5_-edge FIB section 2B) cluster analysis was performed, and this again was done using *aXis2000*. For cluster analysis on oxygen *K*-edge data, the pre-edge region 500 to 528 eV was fit with a line, which was then subtracted from the data. Oxygen *K*-edge data were then normalized to an edge step of 1 by fitting a line to data points above the absorption edge (550 to 600 eV) and ‘flattened’ by subtracting the difference between this line, which is allowed to have a non-zero slope, and unity. Ce *M*_4,5_ and U *N*_4,5_ data were background subtracted using pre-edge data points, and when necessary data points between the two peaks of these spectra. Spectra were then normalized to a peak intensity of 1.

For some oxygen *K*-edge data, where examination of differences in oxygen species from one pixel to the next was possible, data were analyzed using data processing techniques implemented in Python. First, the spectrum for each pixel had a linear background subtracted from 500 to 528 eV. Next, the edge-step intensity for each pixel was determined by fitting a line to data above the absorption edge (550 to 600 eV) and finding where this line crosses 530.5 eV. The choice of 530.5 eV is the approximate average of the inflection point energies of the different clusters identified in FIB section 1B, representing the extent of variation in oxygen spectra in these samples. A line was used rather than a quadratic fit because of high noise in many single-pixel spectra, particularly in thinner regions of the sample, and a higher-order polynomial could introduce significant distortions to the spectra from this noise. For better alignment of these spectra with those in the literature, the spectra were ‘flattened’ by subtracting the difference between this fitting line (with non-zero slope) and the edge-step intensity for points above 530.5 eV. This adjustment was minor, with almost no effect near the edge step where the resulting analysis is performed. Single-pixel spectra were not normalized to an edge step of 1 to prevent the necessity for separate treatment of pixels with no oxygen response.

**Figure 1:**
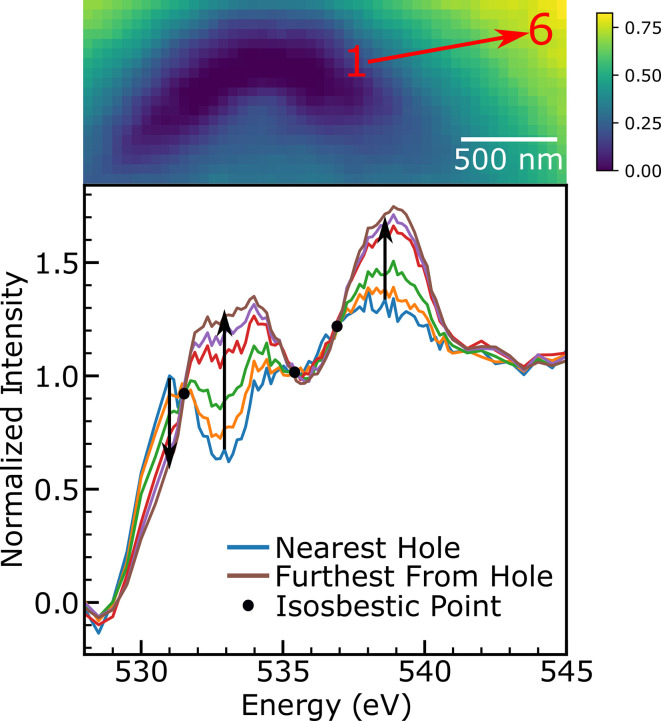
(Top) An optical density image of the stack region on FIB section 1B, sourced from the rim of the pellet, taken at 535 eV (pixel size 50 nm). The red numbers indicate the approximate location of the first and last regions from the central hole. (Bottom) Spectra taken from six different regions (determined by cluster analysis) on FIB section 1B. The blue trace is nearest to a defect in the sample, the intermediate regions are orange, green, red and purple in order of increasing distance from the defect, and the brown trace is furthest from the defect in the sample. The arrows indicate how the spectra change with increasing distance from the defect. These spectra show isosbestic points (black circles), which are a clear indicator of a mixture of only two different oxygen species. Isosbestic points are located at 531.5, 535.3 and 536.9 eV.

Once the stacks were aligned, normalized to *I*_0_ and background subtracted, it became clear that differences in uranium oxide stoichiometry could be resolved at the O *K*-edge and further analysis became necessary. The region showing the largest difference in spectrum (FIB section 1B) was analyzed with *k*-means cluster analysis and showed isosbestic points (Fig. 1 ▸), indicating the presence of only two distinct oxide species (Vinokurov & Kankare, 1998 ▸). Isosbestic points are points where these two oxide species have the same absorption, and so any linear combination of these two species (properly normalized) will have this same absorption value. Other regions with only subtle changes in oxidation state were not amenable to the same type of analysis. The full extent of these spectra, showing that they are properly normalized, is given in the supporting information.

**Figure 2:**
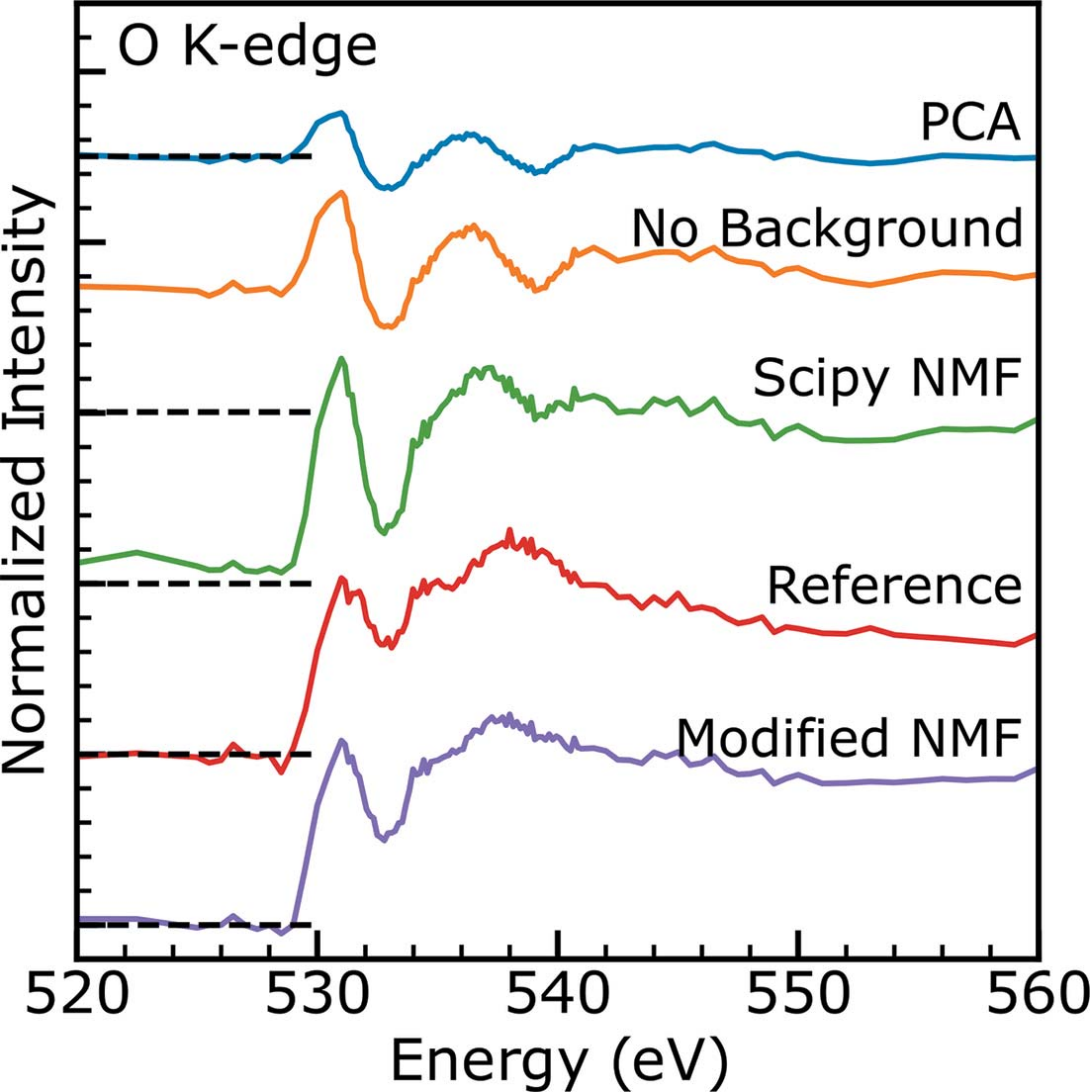
Comparison of the interfacial spectral component for different data analysis methods. PCA (blue) is shown for comparison. NMF without background subtraction (orange), NMF implemented in *SciPy* by removing negative data points (green) and the new modified NMF (purple) methods are compared with the region closest to the defect in Fig. 1 ▸ (red). Dashed lines represent the zero point for each spectrum (note the offset between the orange trace and its dashed line as the background is not subtracted). The modified NMF trace most closely resembles the actual spectrum of the region closest to the defect.

Non-negative matrix factorization (NMF) is a method for algorithmically determining the component pieces of a dataset, while requiring those component pieces to be non-negative (Lee & Seung, 1999 ▸). NMF has been utilized in the past for spectroscopy and even specifically investigated for STXM data analysis (Mak *et al.*, 2014 ▸). However, the data collected on the FIB sections were not entirely non-negative due to noise (after pre-edge subtraction and especially in thin parts of the sample), and so traditional NMF was inapplicable without some modification. Several candidate solutions were investigated and are shown in Fig. 2 ▸. One of the components (later called the interfacial component) is concentrated near the intentional defect where the sample is thinnest, and where the data are noisiest and closest to zero, so the distortionary effects from the modifications to the NMF method are expected to show up in the interfacial component more clearly than in the other component (bulk component). For reference, a similar comparison is done for the bulk component in the supporting information. Each method is constrained to two components (as expected from the isosbestic points above). First, for reference, a principal component analysis (PCA) component is shown. PCA is not ideal for X-ray absorption spectroscopy (XAS) analysis because it allows negative values both in the components and in the weights of those components in the fit, a situation which should be deemed unphysical. In the second example, the background is not subtracted from the data to keep the data positive. However, the resulting component dips below the background much like the PCA component, which is again unphysical. In the third example, negative data values in the background-subtracted spectrum were removed from the data by replacing them with a very small positive value and the NMF analysis was carried out using the *SciPy* library. This has a distortionary effect by raising the pre-edge above zero. The resulting component spectrum for the interfacial component is also subtly different from the closest region to the defect of Fig. 1 ▸ (red trace in Fig. 2 ▸), particularly in the region between the two pre-edge peaks and the low-energy shoulder of the white line. This is not necessarily a problem as the NMF analysis may be un­covering a phase hidden from prior analysis, but this would be surprising given the isosbestic points as revealed in the *k*-means cluster analysis.

The best alternative developed is shown as the final trace in Fig. 2 ▸. This method utilized the original algorithm of Lee and Seung but simply ignored the requirement that the data be non-negative. This could, in principle, lead to negative values for both the components and the weights (the maps), but in at least the case of the data presented here, negative values for the component spectra are confined to the pre-edge and negative weights are confined to areas where no sample is present. This algorithm is reproduced here for reference and discussion (Lee & Seung, 1999 ▸).

Each three-dimensional stack is first reformed into an *m*×*n* matrix (call this *X*), where each of the *n* columns represents the spectrum for a pixel. The goal is to find an approximation *X* = *WH* where *W* is an *m*×*n* matrix of *k* component spectra, each of length *m. H* is a *k*×*n* matrix with each column representing the weights of the component spectra for each pixel. To find these spectra and weights, the *W* and *H* matrices are first initialized with random positive values. Lee and Seung developed update rules for the *W* and *H* matrices,

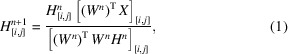




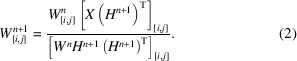

When provided with non-negative data *X* and non-negative initial values, *W* and *H* converge to non-negative values minimizing the difference 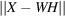 (Lee & Seung, 2000 ▸). In the current implementation, with negative values for some data points, the update rules for *W* and *H* do not always converge for every initialization, but a strong majority do. The hyperstoichiometric component spectrum for stack 1B-1 is shown as the final trace of Fig. 2 ▸. The main difference between the modified NMF method and adjusting the data to be positive (green trace, Fig. 2 ▸) is visible in the pre-edge, which is now centered around zero, and in the shape, which matches more closely the innermost region from the *k*-means cluster analysis which indicated only two oxide species due to the presence of isosbestic points.

Speculatively, because the matrices are updated entry by entry, only in those rows or columns where a substantial number of negative data are present will a negative result for *W* or *H* form. These are either energies (rows) where a large number of negative points are present (the pre-edge which has been subtracted out and therefore has an average of zero), or pixels (columns) where negative values exist for a large number of data points (pixels where there is no optical density). Although this reasoning is not rigorously proven in this analysis, the resulting component spectra, which match so well with uranium oxide standards and high-quality fits (see the supporting information for examples), establish confidence that the modified NMF technique does accurately represent the data. STXM data can often be noisy, particularly at the level of individual pixels, so it is necessary to make some modification to NMF, and this method was deemed to be the least obtrusive.

The data at the oxygen *K*-edge were analyzed using the modified NMF method. Three suitable regions – those with defects which can be aligned – are combined into a single dataset for NMF analysis, so the component spectra are consistent between all regions. Data were fitted over an energy range up to 545 eV. Above 545 eV, focus is lost on some images as a result of zone-plate drift, which introduces significant distortions to the data, particularly around the edge of the sample. Fit components were normalized to an edge step of 1, allowing for quantitative analysis. Because the fit components do not extend beyond 545 eV, normalizing to an edge step of 1 was done by matching these spectra to the cluster spectra collected in Fig. 1 ▸, which do extend into the post-edge and are properly normalized. By normalizing this way, the blurring after 545 eV does not affect the normalization of the NMF components.

The species maps are given in units of edge step which are directly proportional to the area density of oxygen atoms with proportionality given by the scattering cross section, which is tabulated in the Center for X-ray Optics (CXRO) database (Gullikson, 2010 ▸). This area density can be converted to an effective thickness with knowledge of the number density of oxygen in the material. This is not done here to provide a more direct comparison between the spectra of the two differing oxide species present.

### 2.4. Atom probe tomography

Atom probe tomography (APT) analysis (Bachhav *et al.*, 2021 ▸, 2020 ▸; Meisenkothen *et al.*, 2020 ▸) was performed on this fuel to investigate atomic composition from similar regions of the fuel pellet to the STXM FIB sections. Specimens were analyzed at the Center for Advanced Energy Studies (CAES) for APT analysis using a Cameca LEAP 4000XHR. Analysis was performed in laser mode with laser energy in the range of 50 pJ per pulse, a pulse repetition rate of 125 kHz and a data collection rate of 5 atoms per 1000 pulses. The samples were cooled to a base temperature of 50 K. Data analysis was completed with the CAMECA *Integrated Visualization & Analysis Software* (*IVAS*), Version 3.6.8 (https://www.cameca.com/service/software/ivas).

### 2.5. Scanning transmission electron microscopy

A scanning transmission electron microscopy (STEM) image was used to identify features smaller than STXM could resolve. Data used here have been presented in prior work, and the experimental details of these measurements are given in that work (Yuan *et al.*, 2021 ▸).

## 3. Results and discussion

### 3.1. Oxygen *K*-edge X-ray absorption spectroscopy

**Figure 3:**
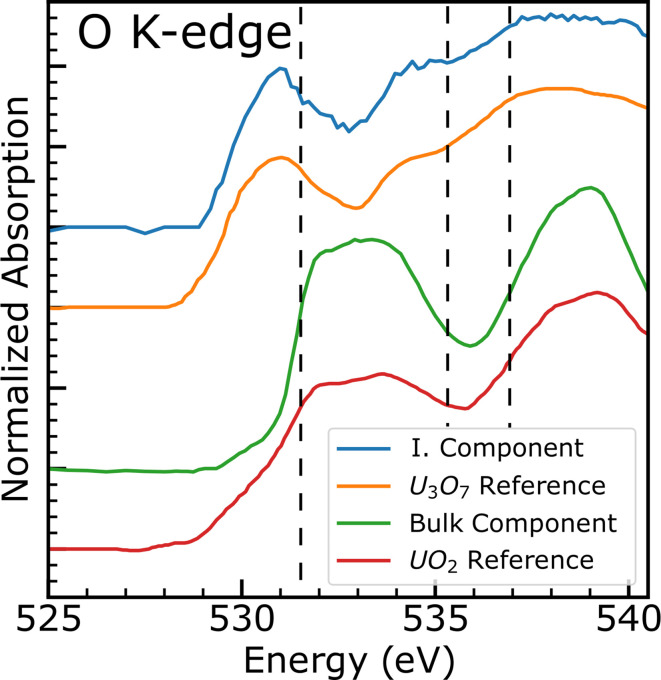
Comparison of the two component spectra obtained by modified non-negative matrix factorization (interfacial in blue and bulk in green) with reference spectra from Conradson *et al.* (2013 ▸) (U_3_O_7_ in orange and UO_2_ in red). Spectra are offset for clarity. The isosbestic points at 531.5, 535.3 and 536.9 eV as determined by *k*-means cluster analysis are shown as dotted lines.

Three stacks taken on three different FIB sections (1B, 2A and 2B) were suitable for the non-negative matrix factorization methods outlined in the previous section. Several other stacks were also collected in different regions but did not have features useful for formally aligning images. The data from the three stacks were processed using the modified NMF technique described in the previous section in a single calculation so that the resulting component spectra would be the same for all three regions. The component spectra and reference ones are shown in Fig. 3 ▸ (Conradson *et al.*, 2013 ▸). The paper containing those reference spectra shows several different O *K*-edge measurements of uranium oxides, but UO_2_ and U_3_O_7_ appear to resemble the component spectra most closely. Conradson *et al.* (2013 ▸) is chosen as a key reference here because it has good qualitative agreement with past work on similar oxides (Denning *et al.*, 2002 ▸; Ward *et al.*, 2016 ▸, 2017 ▸; Pacold *et al.*, 2018 ▸; Tobin *et al.*, 2017 ▸; Jollet *et al.*, 1997 ▸) and has a full range of uranium oxides necessary to evaluate the data collected, though general agreement in the literature on the exact shape of uranium oxide spectra is uneven, with several authors showing different spectra (Magnuson *et al.*, 2006 ▸; Yu *et al.*, 2011 ▸; Wu *et al.*, 1999 ▸). It should be noted that, as seen in the work of Conradson *et al.* (2013 ▸), the spectra of UO_2+*x*_ oxidation states between UO_2_ and U_3_O_7_ are well represented by a linear combination of UO_2_ and U_3_O_7_, so these two components can represent an entire continuum of UO_2+*x*_ oxidation states.

The presence of U_3_O_7_ in this discussion may be surprising given the lower uranium oxidation states typically present in spent fuel (Davies & Ewart, 1971 ▸; Walker *et al.*, 2005 ▸; Kleykamp, 1985 ▸). The main reason why this oxidation state is thought to be present is that, as will be shown in the discussion of FIB section 1B, the FIB sections are likely to be oxidized at their surfaces resulting from exposure to air. The formation of a thin layer of U_3_O_7_ on the surface of UO_2_ under ambient conditions has been observed (Hoekstra *et al.*, 1961 ▸). The reaction of spent fuel with air is more complicated, as the doping of the UO_2_ with plutonium and fission products becomes important (McEachern & Taylor, 1998 ▸; Thomas *et al.*, 1993 ▸). Studies of spent fuel show that oxidation proceeds first along grain boundaries to UO_2.4_, though this state is often described as U_4_O_9+*x*_ given its similarity to the structure of U_4_O_9_ (UO_2.25_) (Thomas *et al.*, 1993 ▸; Einziger *et al.*, 1992 ▸). This U_4_O_9+*x*_ uranium phase has not been studied by O *K*-edge XAS, so U_3_O_7_ (UO_2.33_) may be the closest analog available as it is the closest in stoichiometry to UO_2.4_. Considering that the spectrum of U_4_O_9_ is well represented by a linear combination of those of UO_2_ and U_3_O_7_, it seems reasonable that the U_3_O_7_ spectrum would be a fairly close match to that of U_4_O_9+*x*_.

As will be shown below, one component is concentrated at the interface of the sample with voids or cracks, so this component is referred to as the interfacial component. The use of this term rather than referring to this component as U_3_O_7_ is to convey a degree of uncertainty in the exact composition of this component, given the lack of full agreement between the uranium oxide spectra in the literature and because of the complex nature of spent fuel oxidation following burnup. The other component is present in the bulk of the material and most closely resembles the spectrum for UO_2_.

This two-component fit is compared with a three-component fit in the supporting information so that the possibility of a third component could be investigated. The third component could arise from a small grouping of different uranium oxides, from differing chemistries across the respective FIB sections and/or from experimental differences when measuring different FIB sections. An extensive investigation of this is outside the scope of this research, but this dataset remains a possible source of future insights with much further study.

**Figure 4:**
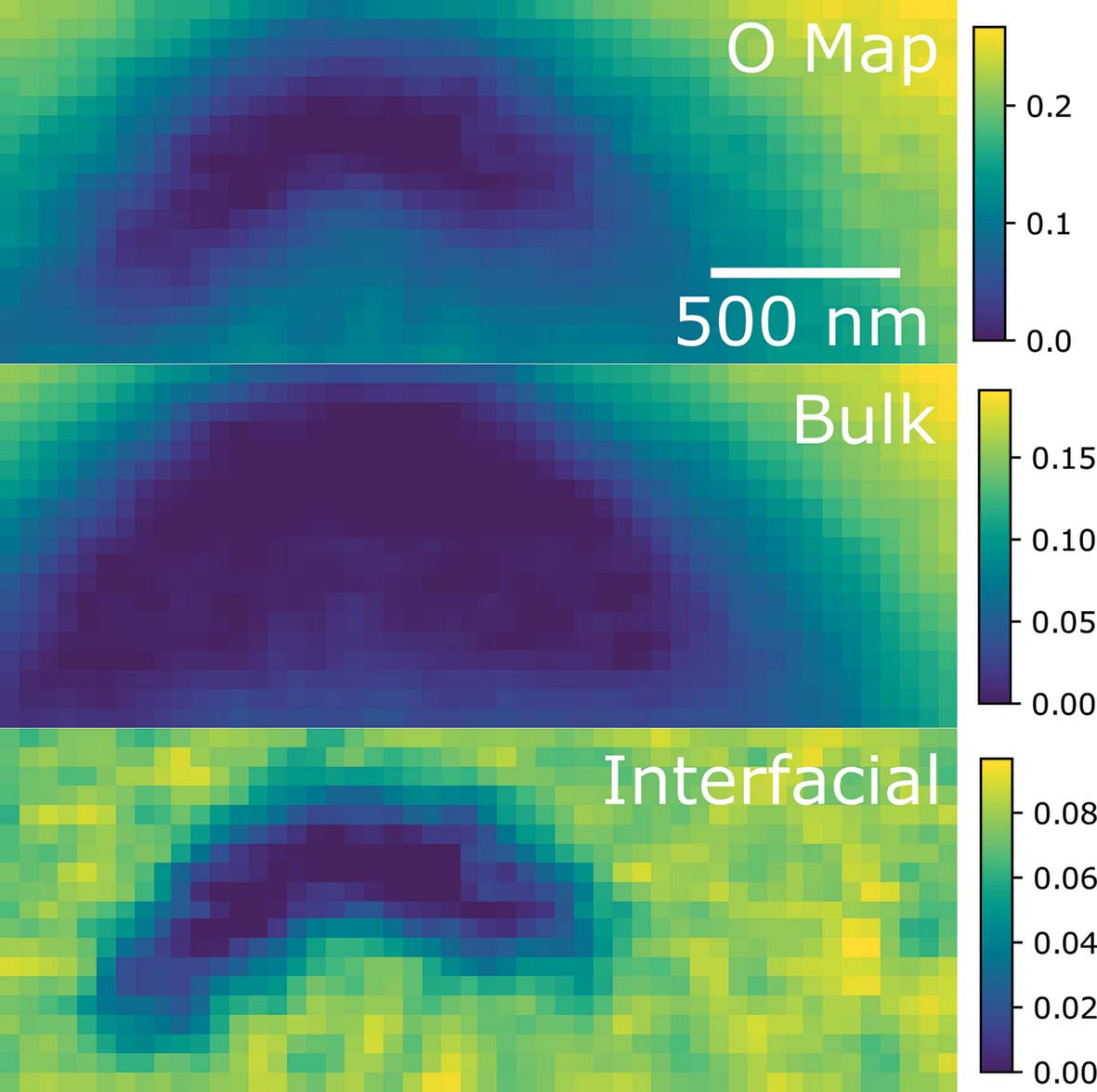
(Top) An oxygen map, and (middle) bulk and (bottom) interfacial species maps of FIB section 1B near the defect in the sample generated by the FIB. Note the separate scale for each map, where the values are normalized to the oxygen edge step (proportional to the total oxygen content in each phase). The STXM pixel size is 50 nm × 50 nm.

The component weights for each component spectrum are shown in Fig. 4 ▸. Because the spectra are normalized to an edge step of 1, these fit coefficients are directly proportional to the amount of oxygen in each phase, represented by the component spectra. This stack was taken from FIB section 1B which was sourced from the rim of the pellet. The crescent-shaped hole in this region was generated by the FIB instrument, so the oxidation observed near this hole gives some indication of sample oxidation following FIB sectioning. The top image of Fig. 4 ▸ is an oxygen map which is roughly proportional to the overall thickness of this region of the FIB section. Near the hole, the bulk UO_2_-like component is completely absent, and the interfacial component is dominant and increases as the sample thickness increases. Further away from the hole, the bulk component increases with the thickness of the sample but the interfacial component remains relatively constant. This is consistent with the interfacial component being a thin layer over the sample and the bulk material remaining largely pristine inside this thin layer. One interesting feature is that the mottled texture shown in the interfacial map does not result from noise, but rather from real differences in the data. Examples of single-pixel spectra show substantial differences between neighboring pixels and the fit of the modified NMF method is able to pick these differences up. Some single-pixel spectra are shown in the supporting information for reference. Grain boundaries and small voids from fission product precipitates are not expected to be visible at the resolution achieved by STXM (tens of nanometres).

**Figure 5:**
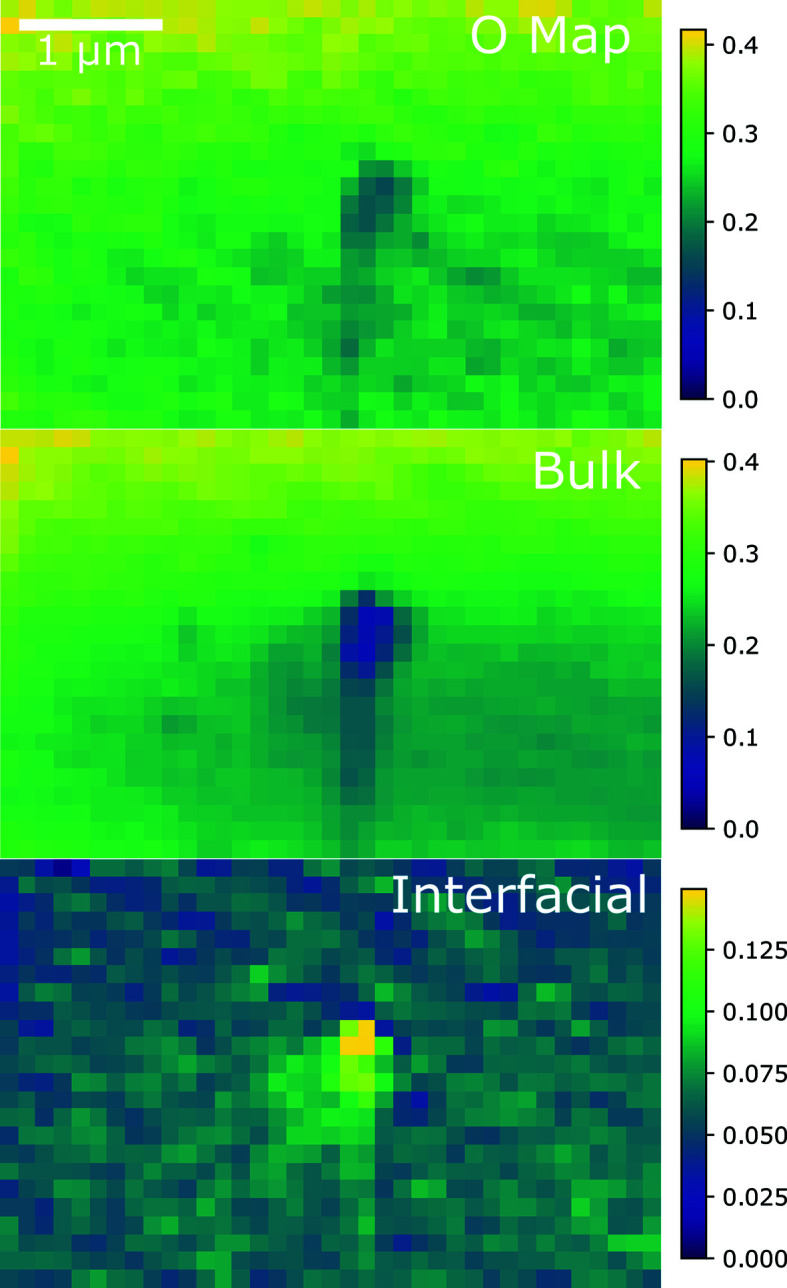
(Top) An oxygen map, and (middle) bulk and (bottom) interfacial species maps of FIB section 2A (sourced from the pellet’s center) near a defect in the sample. The color bars on the right-hand side indicate a quantitative measure of the oxygen content in each phase, with the scale corresponding to the oxygen edge step for each pixel. The STXM pixel size is 100 nm × 100 nm.

The species maps of a stack from FIB section 2A are shown in Fig. 5 ▸ together with an oxygen map for reference. This section was sourced from the center of the pellet. The hole here was not generated by FIB but is instead an actual defect in the original fuel pellet. A similar pattern is seen here to FIB section 1B, which showed increased oxidation at the surface of the sample. Here this is visible even more strongly where the hole in the sample is highly oxidized, but there remains a thin layer of oxidation over the rest of the sample. It is hard to distinguish here whether the oxidation shown by these maps results from oxidation after FIB sectioning or while the pellet was undergoing burnup. If oxidation occurs more rapidly around defects, this would be difficult to distinguish from oxidation after the fact.

**Figure 6:**
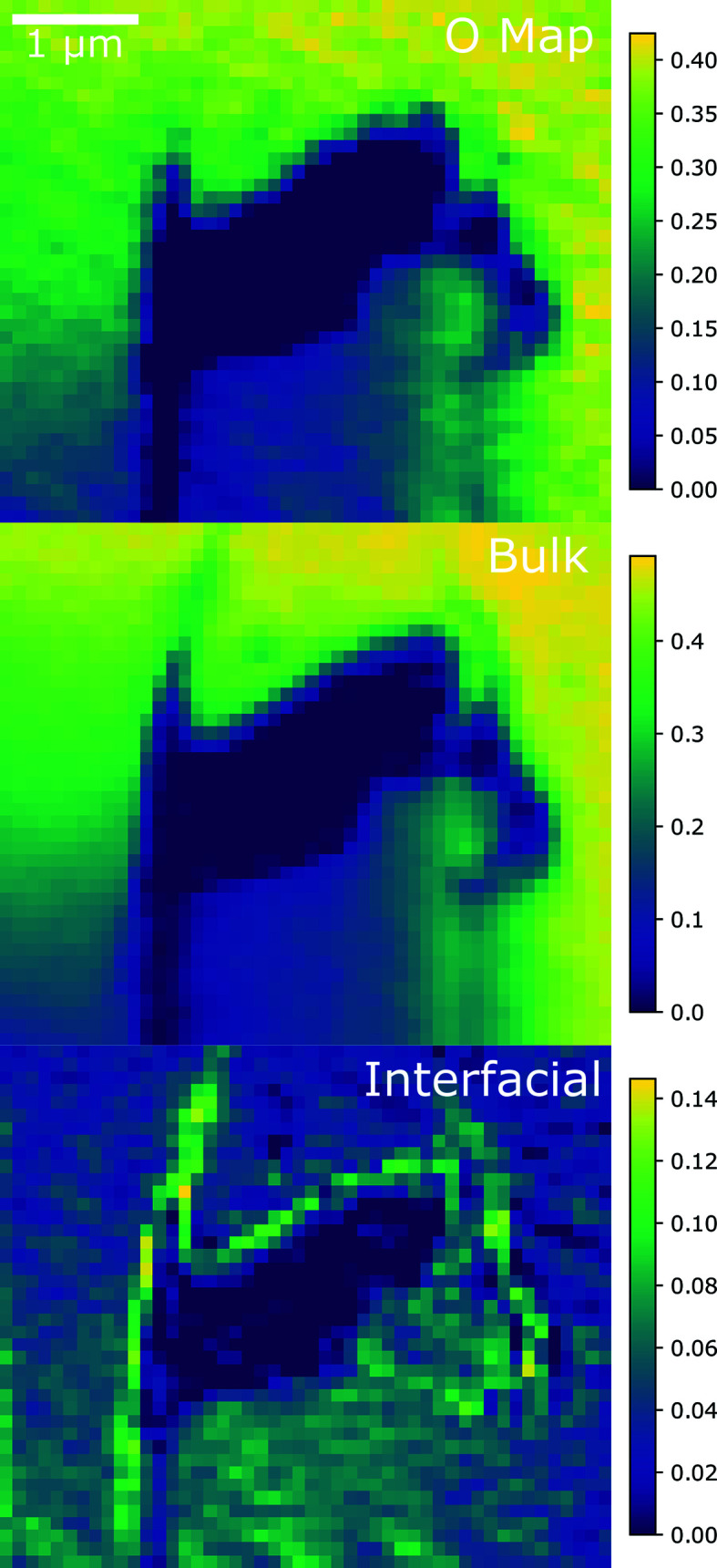
(Top) An oxygen map, and (middle) bulk and (bottom) interfacial species maps of FIB section 2B near a large defect in the sample, sourced from the center of the pellet. The color bars on the right-hand side indicate a quantitative measure of the oxygen content in each phase. The STXM pixel size is 100 nm × 100 nm.

The component maps for the final stack analyzed with NMF are shown in Fig. 6 ▸. This stack was taken on sample 2B, sourced from the center of the pellet near a large defect present in the fuel. Like the others, these maps show a constant layer of hyperstoichiometric oxide throughout the sample away from defects. In the lower part of this map, there is a thin region in the overall sample, and the bulk component increases and decreases with the overall thickness of the sample, whereas the interfacial component remains essentially constant. However, the interfacial component is concentrated along the edge of the defect. This large concentration of a higher oxide is expected near a defect of the sample, if a small layer of the surface of the sample has been oxidized.

**Figure 7:**
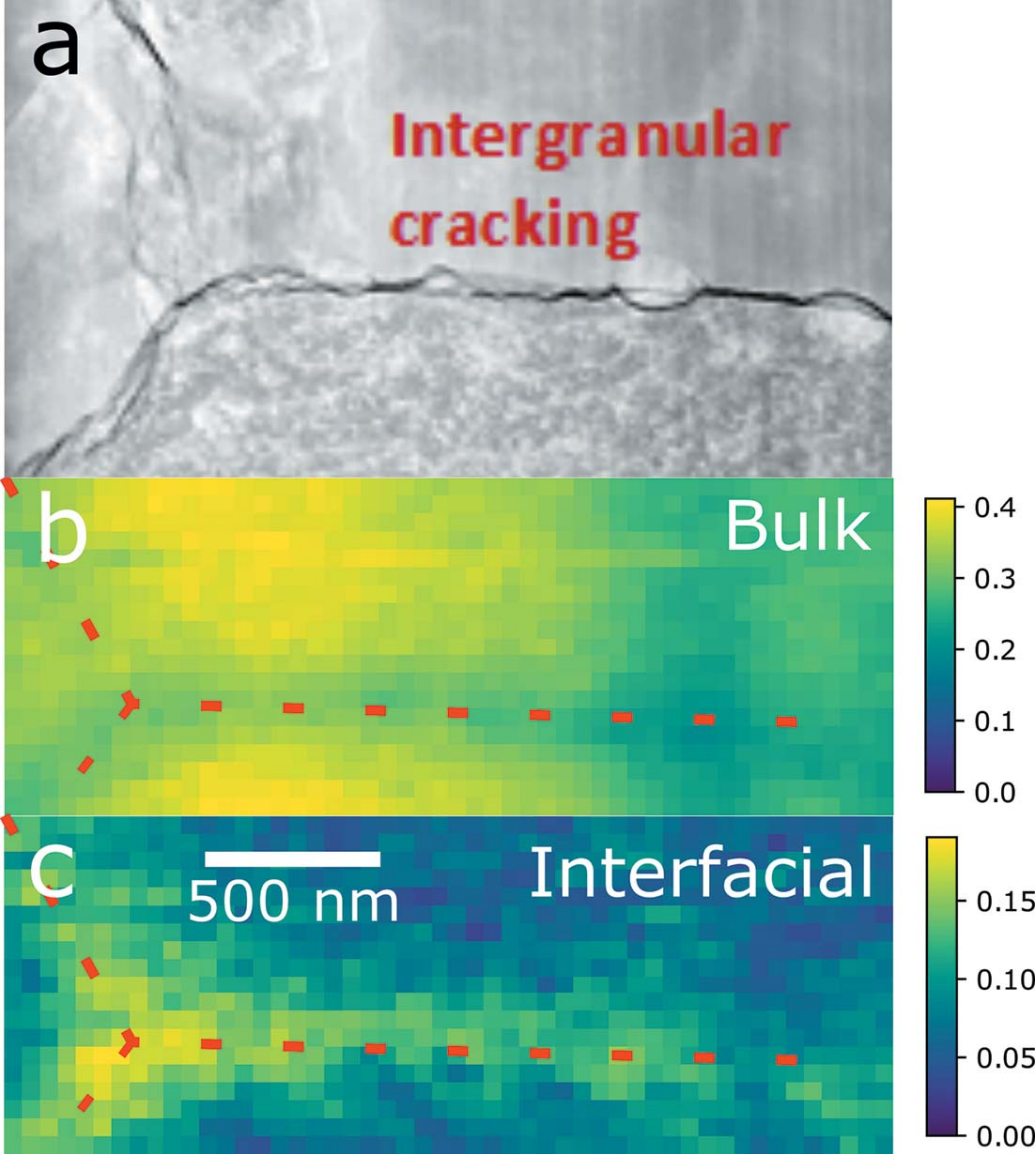
A comparison of (*a*) a high-angle annular dark-field (HAADF) scanning transmission electron microscopy (STEM) image with (*b*) bulk and (*c*) interfacial species maps of sample 1A, taken from the rim of the pellet. Each pixel of the stack was fitted as a linear combination of the NMF component spectra. Red lines on the species maps are a guide for the eye to indicate the position of the intergranular crack. Note the difference in the maxima of the intensity scale bars on the right-hand side. The STXM pixel size is 50 nm × 50 nm and the HAADF-STEM image is scaled to match the STXM images.

Finally, Fig. 7 ▸ shows a similar map for a region of the sample taken from the rim of the pellet which was also imaged using TEM. Because of the lack of features suitable for alignment, this stack had to be aligned manually. The stack was also truncated in energy so that the drift of the zone plate did not remove the features of interest. Because of these factors, this stack was not analyzed using NMF. Instead, the NMF components generated with the other stacks were used to fit the spectrum for each pixel to generate a component map similar to Figs. 4 ▸–6 ▸ ▸. The resulting component map shows a large contribution from the interfacial component near the intergranular crack visible in the STEM image [Fig. 7 ▸(*a*)]. The areas away from the crack predominantly consist of the bulk component. It is also noteworthy that additional oxidation is not observed near the smaller grain boundaries visible in the TEM image, whereas additional oxidation is evident near the large crack.

Taken together, the spectra from each FIB section suggest a thin layer of hyperstoichiometric oxide has formed over the surface of these samples, which includes both the front and back surfaces of the sample, along with the interface between the sample and cracks or defects. All of the features in each of the component maps shown are supported by this hypothesis, and this is consistent with how uranium is known to oxidize (Spurgeon *et al.*, 2019 ▸; Thomas *et al.*, 1993 ▸; Einziger *et al.*, 1992 ▸). The fairly constant presence of the interfacial component away from defects, but regardless of sample thickness, and the increased concentration of this component at interfaces with voids or cracks in the sample both support this model. This component most closely resembles U_3_O_7_ by comparison with reference spectra, although there is variation in the literature O *K*-edge spectra of uranium oxides, and the complexity of spent fuel oxidation makes unequivocal assignment difficult. The area around the hole in sample 1B is shown to be entirely interfacial uranium oxide with no bulk UO_2_-like component present. Other areas are more ambiguous with a mixture of the bulk and interfacial components present. It is possible to construct a spectrum similar to U_4_O_9_ and other UO_2+*x*_ oxides with a linear combination of UO_2_ and U_3_O_7_ spectra, so it is not possible to distinguish between a thin layer of U_3_O_7_, a layer of U_4_O_9_, and mixtures thereof with stoichiometric UO_2_ (Conradson *et al.*, 2013 ▸). The other limitation to this technique is that linear combinations of these component spectra would give identical fits. This means that the bulk and interfacial components do not necessarily correspond to the physical presence of those particular uranium stoichiometries in the sample.

A quantitative estimate can be made for the thickness of this layer if its density is known. For this purpose, U_3_O_7_ was chosen as the interfacial component. This is done by using the calculated penetration depth of U_3_O_7_ at X-ray energies just above and below the oxygen *K*-edge to determine the thickness required to get an edge step of 0.055, which is the average of the interfacial component away from any defects in the three FIB sections for which NMF analysis was performed. This results in an oxide layer of 16 nm, or 8 nm on each face of the FIB section. This is consistent across the different FIB sections, whether it be from the center of the pellet or from the rim. Uncertainty in these values is probably driven by systematic differences in background subtraction and normalization in the analysis of these data, rather than statistics and noise in the data, and is therefore difficult to estimate accurately.

Comparing the oxidation state from the center of the pellet to the rim could be interesting, but is complicated because of the presence of the layer of hyperstoichiometric oxide formed on the surface. Away from defects, no discernible difference is observed between samples sourced from the rim and the center of the pellet. One reason this difference may not be visible is that if the oxidation is driven by the partial pressure of oxygen at the surface of voids and cracks in the pellet during burnup, it would not be possible to distinguish this from oxidation due to air, as those areas were also exposed to air during sample transportation and storage.

### 3.2. Uranium *N*_4,5_-edge and barium *M*_4,5_-edge

**Figure 8:**
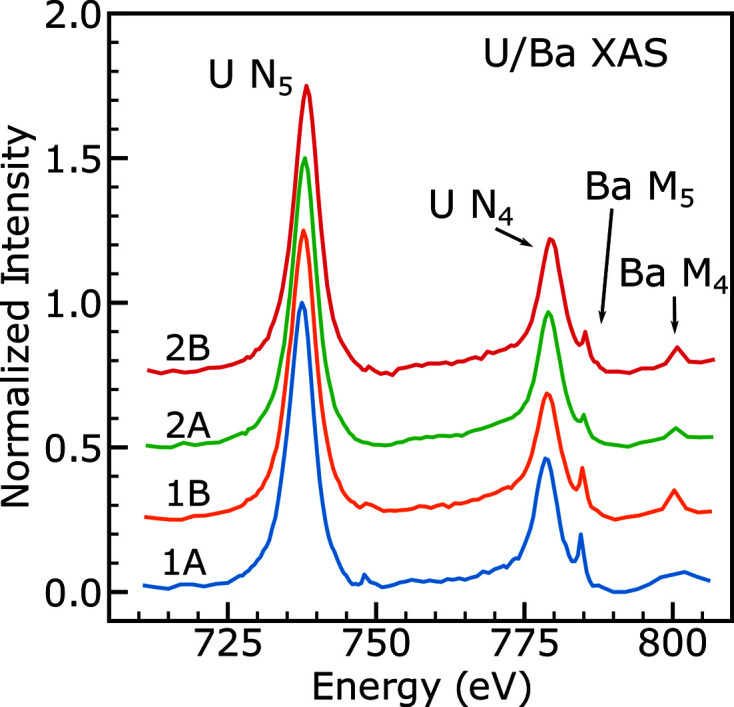
A comparison of U spectra on four different FIB sections in the sample. 1A and 1B are located on the rim of the pellet, and 2A and 2B are located near the center. Each spectrum is normalized to a U *N*_5_ peak intensity of 1. Spectra are offset by 0.25 for clarity.

Spectra at the U *N*_4,5_ and Ba *M*_4,5_-edges were collected on all four FIB sections. The average U/Ba spectrum in each location is shown in Fig. 8 ▸. The U *N*_4,5_ spectrum consists of two main peaks, the *N*_5_, an excitation of the 4*d*_5/2_ electrons, at 737.0 eV, and the *N*_4_, an excitation of the 4*d*_3/2_ electrons, at 778.8 eV. The single white line at these absorption edges is typical of the U *N*_4,5_-edge, with few other distinguishing features (Tobin, 2014 ▸).

**Figure 9:**
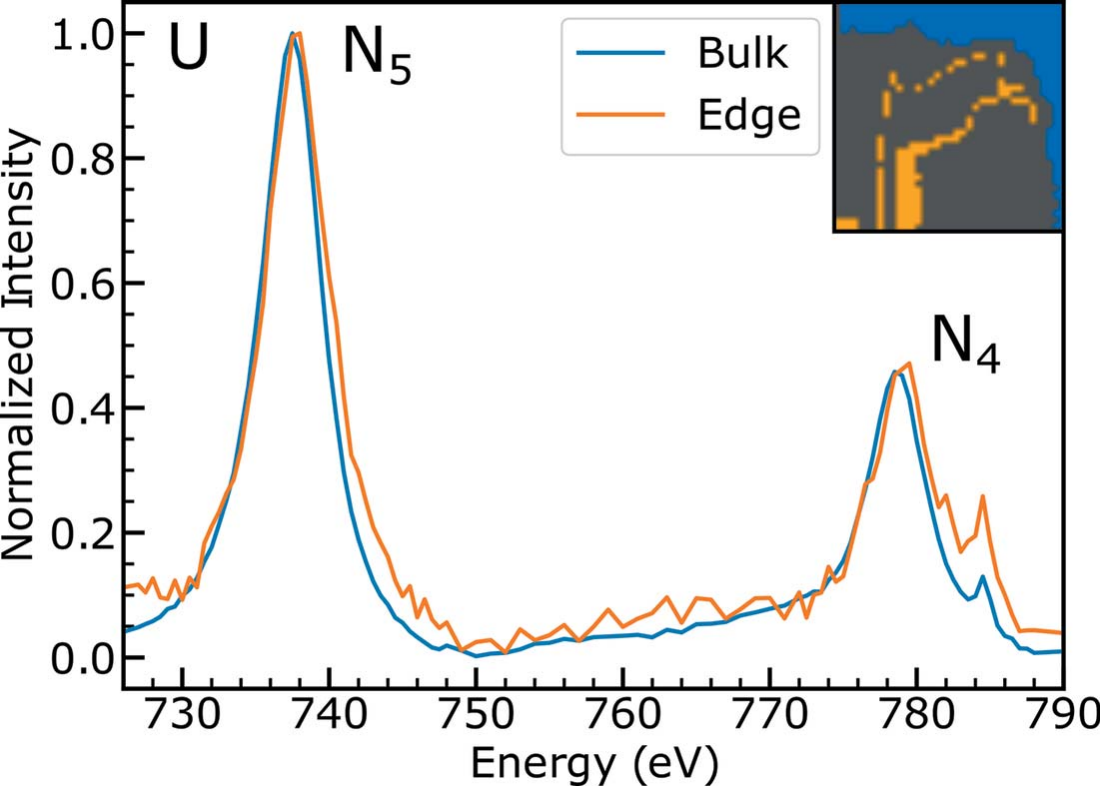
Uranium spectra on sample 2B (same location as Fig. 6 ▸). The inset shows the two regions the averaged spectra were taken from. The interfacial spectrum has peaks slightly higher in energy, as expected for a higher oxidation state of uranium.

Uranium compounds with different oxidation states have a different peak energy and peak intensity, but the type of species-specific information available at the O *K*-edge is not present at the U *N*_4,5_-edge. The U *N*_4,5_-edge is not highly sensitive to oxidation state, and this result is only mentioned here as a confirmation of the more quantitative O *K*-edge data (Tobin *et al.*, 2015 ▸; Tobin, 2014 ▸). In the previous section, the sample was shown to be oxidized around the edge of the sample and near defects. Fig. 9 ▸ shows cluster analysis of one U *N*_4,5_ stack taken of FIB section 2B. The most oxidized regions (orange) have a very slightly higher peak energy (difference of 0.25 eV at the *N*_5_-edge) compared with the bulk regions (blue). Referring to the literature, this shift to higher energy indicates a higher oxidation state (Tobin *et al.*, 2015 ▸). This shift is consistent in other datasets across other FIB sections where the most oxidized region shows a slightly higher peak energy in the U *N*_4,5_ spectrum.

The Ba *M*_4,5_-edge is not a focus of this work, and thus few data points were taken over those edges. However, some interesting results can still be drawn from these few data points. By looking at the Ba peak intensities in Fig. 8 ▸ relative to the U peak intensities, the overall concentration of Ba is determined to be higher at the rim of the pellet (samples 1A and 1B) than at the center (samples 2A and 2B), as is expected from prior work (Sari *et al.*, 1979 ▸). Barium typically forms a barium zirconium oxide which is a solid precipitate in nuclear fuel. A Ba map can be generated by subtracting two of the images in the stack, and Ba is found to be evenly spread throughout the individual FIB sections, with one notable exception in the interior of the pellet (holder 2). The general trend of a homogenous distribution of Ba in the lamellae is not inconsistent with Ba precipitating out of the UO_2_ matrix, as long as these precipitates are too small for STXM to resolve. FIB section 2B shows one area of higher Ba concentration, which is discussed further in the supporting information. Comparing oxygen spectra in the measurable Ba inclusion and outside of this Ba inclusion shows no real difference, indicating that the Ba content is not high enough, even in this inclusion, to change the oxygen spectrum significantly.

### 3.3. Cerium oxidation state

**Figure 10:**
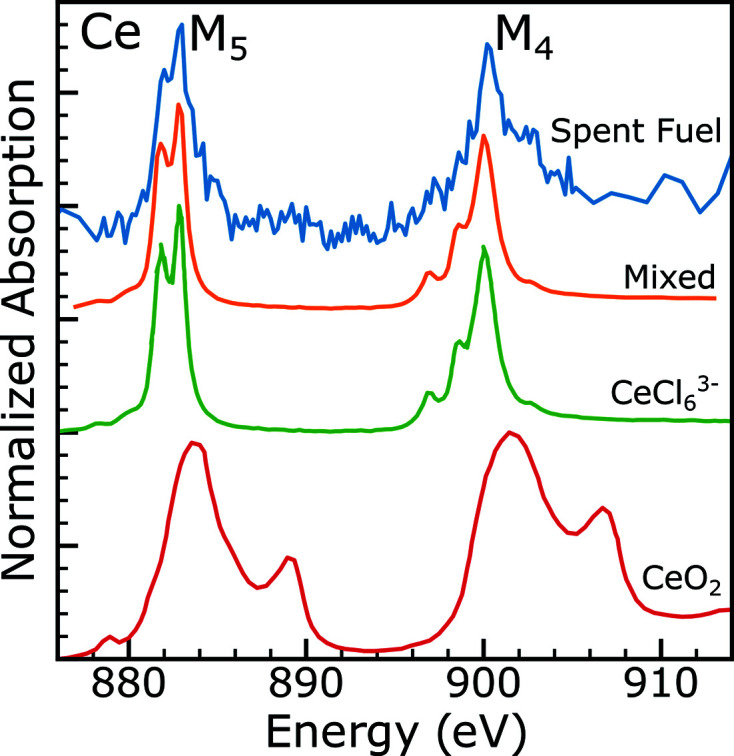
Cerium *M*_4,5_-edge spectra for the spent fuel FIB section 2B (blue) sourced from the edge of the sample, trivalent CeCl_6_^3−^ (green) and tetravalent CeO_2_ (red). Trivalent and tetravalent reference spectra are taken from Smiles *et al.* (2020 ▸). Also shown is a mixed reference (orange), generated by a linear combination of 80% CeCl_6_^3−^ and 20% CeO_2_.

Spectra at the Ce *M*_4,5_-edge were taken on two FIB sections sourced from the center of the pellet (samples 2B and 2A). A representative Ce spectrum, from sample 2B, is shown in Fig. 10 ▸. The Ce spectrum shown is quite noisy, due to the low amount of Ce in the sample and the limited beam time available to collect the Ce spectra. The *M*_5_-edge consists of a pair of peaks at approximately 882 and 882.7 eV, representing an excitation of 3*d*_5/2_ electrons. The *M*_4_-edge consists of a single peak at approximately 900 eV and a shoulder at 902 eV, representing the excitation of 3*d*_3/2_ electrons. The two regions measured both yielded similar spectra with these same features, and a comparison of those spectra is available in the supporting information. Cerium maps generated from the stacks collected show no measurable difference in Ce concentration throughout the sample. Estimation of total Ce content is difficult given how the peak intensity is so large compared with the edge step, and so this quantification is left to APT in the next section.

Fig. 10 ▸ also shows representative Ce^3+^ and Ce^4+^ spectra (Smiles *et al.*, 2020 ▸). Ce_2_O_3_ would be a better representation of trivalent cerium in this sample, but the differences between different trivalent cerium spectra are very subtle and would not change the analysis presented here. The data collected on the spent fuel FIB section show many of the features distinct to a Ce^3+^ spectrum. The two peaks of the *M*_5_-edge, at 881.8 and 882.8 eV, are characteristic of trivalent cerium. The other distinctive feature of trivalent cerium is the three ‘sawtooth’ peaks on the low-energy side of the *M*_4_-edge, which are explained by complex multiplet effects as has been demonstrated (Thole *et al.*, 1985 ▸). Because of the low cerium content in these samples, the data quality is not high enough to resolve these ‘sawtooth’ peaks. Notably absent from these spectra are the high-energy satellites present in tetravalent cerium at 889.5 and 907 eV. There is perhaps a slight contribution to the spent fuel spectrum from tetravalent cerium, shown by the high-energy shoulder of the *M*_5_ peak and a slight bump in intensity around 890 eV. A comparison with an 80% trivalent/20% tetravalent combination generated from the reference spectra shows a good reproduction of those features. However, due to the low cerium content, a quantitative measure of the amount of cerium is not possible at this time.

Cerium is easily incorporated into the structure of UO_2_ and the resulting oxidation state of cerium has been of considerable interest (Markin *et al.*, 1970 ▸; Hanken *et al.*, 2011 ▸; Griffiths *et al.*, 1994 ▸; Eloirdi *et al.*, 2018 ▸; Antonio *et al.*, 1996 ▸; Tracy *et al.*, 2015 ▸). It has been suggested that uranium and cerium can transfer electrons as U^4+^ + Ce^4+^ → U^5+^ + Ce^3+^ (Griffiths *et al.*, 1994 ▸). This hypothesis is consistent with the oxidation state measurements made here at the Ce *M*_4,5_-edges and the uranium oxidation state measurement made at the O *K*-edge, which showed the presence of higher uranium oxidation states. It should be noted that because the cerium content of these samples is small, the oxidation of uranium from cerium is expected to be a small effect compared with other pathways of uranium oxidation like reaction with air and water. DFT calculations have shown that this charge transfer is energetically unfavorable but can be entropically stabilized at high temperatures (Hanken *et al.*, 2011 ▸), suggesting that the cerium oxidation state could depend on the thermal history of the sample. Thin-film X-ray photoelectron spectroscopy studies have shown that cerium reduces in the presence of uranium and uranium oxidizes in the presence of cerium (Eloirdi *et al.*, 2018 ▸). Furthermore, high-energy heavy-ion radiation has been shown to reduce CeO_2_ (Tracy *et al.*, 2015 ▸; Pakarinen *et al.*, 2015 ▸). On the other hand, XANES measurements on Ce_2_UO_6_ at the Ce *L*_3_-edge showed no presence of Ce^3+^ (Antonio *et al.*, 1996 ▸). This current study of spent nuclear fuel is not necessarily inconsistent with that prior XANES result as these are very different sample environments and, in particular, very different cerium concentrations. These prior results indicate that this measurement of the oxidation state of cerium in spent nuclear fuel could be an important addition to general knowledge of cerium chemistry in spent fuel.

Cerium at the interface with the lamella could be oxidized from exposure to air following FIB sectioning or be reduced resulting from surface termination effects or heavy-ion radiation. This has been seen in X-ray photoelectron spectroscopy studies of thin films of CeO_2_ (Mullins, 2015 ▸) and XAS studies of Ce–Gd oxide nanoparticles (Eriksson *et al.*, 2018 ▸). Because the sample is relatively thin, the surface could contain a substantial fraction of the cerium in the sample, depending on how deeply the oxidation or reduction occurs. Assuming the model of a thin hyperstoichiometric layer of oxide forming over the surface of the FIB section, the O *K*-edge results collected on this region of FIB section 2B indicate that approximately 16 nm of the sample is the hyperstoichiometric oxide phase and 130 nm is the bulk phase in this region of the sample. Assuming the hyperstoichiometric oxide is forming on the surface of the sample, the Ce measurements are consistent with tetravalent Ce in the hyperstoichiometric region and trivalent Ce in the bulk of the material, but the current measurements cannot distinguish this from a small amount of tetravalent Ce spread evenly throughout the sample. This is consistent with prior XPS measurements on sputtered uranium oxide and cerium oxide films (Eloirdi *et al.*, 2018 ▸). Eloiridi and co-workers showed that Ce^IV^ was formed with U^V^ or U^VI^, but not with U^IV^, so the presence of Ce^IV^ only in the oxidized layer is expected based on those results. What is indisputable from these data is that Ce is in a majority tri­valent state, in a matrix of predominantly UO_2_.

### 3.4. Atom probe tomography

**Figure 11:**
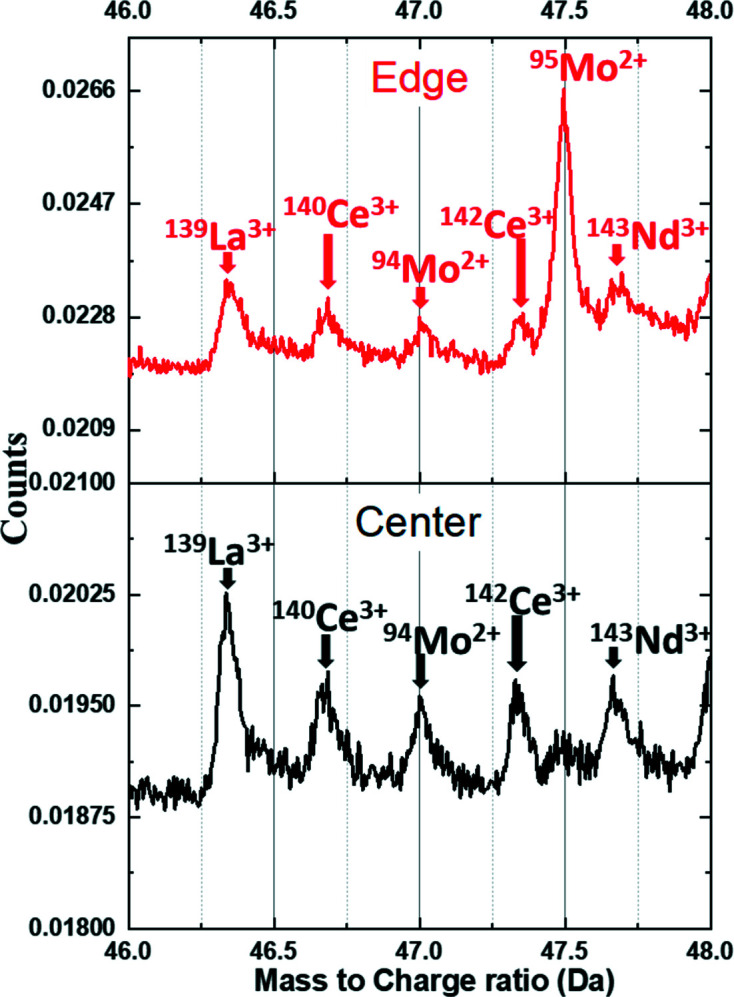
Mass spectra of UO_2_ fuel for specimens prepared from the center of the pellet (in black) and on the rim (in red) using APT, showing ionic species of ^140^Ce^3+^ and ^142^Ce^3+^.

To confirm the presence of Ce in the sample, complementary atom probe tomography (APT) analysis was performed on FIB sections taken from similar regions of the pellet to the STXM FIB sections, near both the center and the rim of the sample. Mass spectra showing a signal from Ce^3+^ for specimens prepared from both the center and rim locations of the fuel are shown in Fig. 11 ▸. Since APT is based on field evaporation of ions from the apex of the specimen, it does not have the ability to determine the valence state of species, as this evaporation can change the oxidation state of the evaporated atoms. For instance, APT can confirm the presence of U and O which field-evaporate predominantly as UO_2_^1+,2+^, UO^1+,2+^, O^+^ and O_2_^1+^ but does not provide information on the valence state of U in the original specimen. More detailed studies of burnup assessment and its correlation with fission products are planned for future publication but are beyond the scope of the current work. The 3D distribution of Ce in the analyzed volume is shown in the supporting information for specimens prepared from the center and rim locations. Both reconstructions show a uniform distribution of Ce ions similar to the uranium molecular ions (UO_2_^1+,2+^, UO^1+,2+^, O^+^ and O_2_^1+^) with total concentrations ranging between 0.02 and 0.03 at.%. Due to the closeness of the peaks in this region of the spectrum, this quantification is complicated. This uniform distribution of Ce is expected given Ce is part of a solid solution with the UO_2_ matrix, and this result is consistent with Ce STXM measurements. The possible presence of CeO ions (^140,142^Ce^16^O^1+,2+^) in the mass spectrum is difficult to confirm due to overlap with the fission product gadolinium (Gd) which has isotopes at 154, 155, 156, 157, 158 and 160, further complicating quantitative analysis. However, the mass spectrum does show distinct peaks for Ce^3+^, which complements the soft X-ray spectromicroscopy findings. The low concentration of Ce in the sample shows that Ce *M*_4,5_-edge STXM is a particularly sensitive measurement tool for identifying the Ce oxidation state in spent fuel.

## 4. Conclusions

Soft X-ray spectromicroscopy data at the O *K*-edge, U *N*_4,5_-edges and Ce *M*_4,5_-edges were collected for the first time on spent nuclear fuel FIB sections. Analysis yielded two component spectra, one concentrated at the interface of the sample near holes, defects and an intergranular crack, and one spread through the bulk of the sample. Matching to reference spectra showed the interfacial component to be a hyperstoichiometric UO_2+*x*_ species and the bulk component to be UO_2_. The resulting component maps are well explained by the model of a thin layer of oxide on the surface of the sample, most likely due to oxidation of the samples in air after FIB sectioning. Analysis of regions of the sample far from defects indicates that this layer is approximately 8 nm thick. This surface oxidation layer was consistent across samples taken from the center and rim of the pellet and so no difference in uranium oxidation between the center and the rim was observed. Based on these results, future O *K*-edge measurements of key intermediate uranium oxides between UO_2_ and UO_3_ would serve to lessen the uncertainties in future studies.

The cerium oxidation state was also measured on a sample sourced from the center of the pellet at the Ce *M*_4,5_-edge. Ce was determined to be 0.5 ± 0.1 wt%. The cerium oxidation state was determined to be at least majority trivalent cerium, with the remainder being made up by tetravalent Ce, but a completely trivalent cerium oxidation state cannot be ruled out due to the difficulty in measuring a low Ce concentration in an absorbing matrix. The possible tetravalent contribution is potentially explained by the hyperstoichiometric layer determined from the O *K*-edge work. These results are in agreement with prior *ex situ* work on model systems and represent an important contribution as the first STXM measurements of the Ce oxidation state in spent nuclear fuel.

A modification to NMF, *i.e.*, taking out the requirement that the data be non-negative, was developed to treat the oxygen spectra, resulting in quality fits to the data without the distortion of other methods explored for data analysis. A number of improvements to this method could be implemented in the future. For example, Mak *et al.* (2014 ▸) laid out several improvements to Lee and Seung’s algorithm, including weighting for sparsity and closeness to target spectra. These improvements are relatively simple to implement, but this was not deemed necessary for this dataset, and it was unclear how these changes would interact with the removal of the requirement for non-negative data. Other, more recent, algorithms for NMF also exist, including a machine-learning improvement designed for STXM analysis, but exploring these fully is beyond the scope of this experiment (Kim *et al.*, 2014 ▸; Shiga & Muto, 2019 ▸).

FIB sectioning represents an attractive method for sample preparation for STXM analysis due to the ideal thickness for soft X-ray spectroscopy and the low amount of material required. The next steps in this development include a wider variety of sample types, such as advanced fuels like uranium nitrides and silicides, mixed oxide fuels and plutonium oxides. This work will require a strong coupling with theory, as a wide library of X-ray spectra of these materials is not available. Another useful avenue of work could be expansion into ptychography, which could allow for the resolution of finer features such as fission product precipitates. This work has shown that STXM of FIB sections is a valuable technique for the study of nuclear fuels and other highly radioactive mater­ials.

## Supplementary Material

Additional details. DOI: 10.1107/S1600577521012315/yx5003sup1.pdf
