# Supplementary material for: Chemical and elemental mapping of spent nuclear fuel sections by soft X-ray spectromicroscopy
Source: J Synchrotron Radiat. 2022 Jan 1;29(Pt 1):67–79. doi: 10.1107/S1600577521012315 (PMC8733983; doi:10.1107/S1600577521012315)
Supplement: Supplementary file 1 [file s-29-00067-sup1.pdf]

JOURNAL OF SYNCHROTRON RADIATION

Volume 29 (2022)

Supporting information for article:

Chemical and elemental mapping of spent nuclear fuel sections by soft X-ray spectromicroscopy

Alexander Scott Ditter, Danil E. Smiles, Daniel Lussier, Alison B. Altman, Mukesh Bachhav, Lingfeng He, Michael W. Mara, Claude Degueldre, Stefan G. Minasian and David K. Shuh

S1. Bulk Component Comparison

For completeness, a similar comparison of NMF modification methods as described in the main text in section 2.3 and Fig. 2 is shown here for the "bulk" component. Here there are a few notable differences. The PCA component is the average component and so looks like an XAS spectrum. The green trace does not have the same problems as the component in Fig. 2. This is because this bulk component is mostly located in thicker sections of the sample, and so removing the negative values has little effect on the data as there are fewer negative values and these values are of a lower relative magnitude following background removal. The modified NMF method (purple) is very similar, indicating that the modifications to NMF do not break the part of standard NMF that is already working.

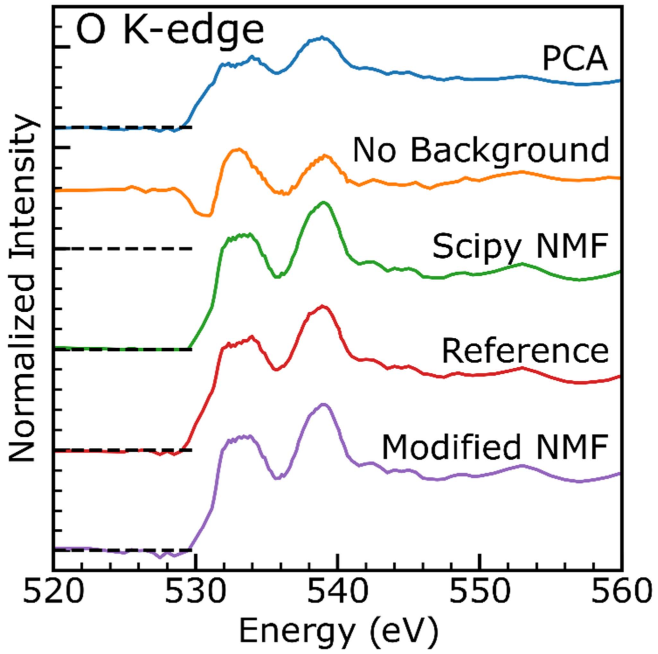

Figure S1 Comparison of PCA (blue), NMF without background subtraction (orange), modification of NMF implemented in scipy to remove negative data values (green), a bulk-like reference from stack 1B (red), and the component generated by the modified NMF method described in the text (purple). Dashed lines represent the zero value for each spectrum.

S1. Number of Components for Modified NMF

The number of components utilized in NMF is a free parameter, able to be adjusted. Our analysis of the isosbestic points of Fig. 1 shows that there are only two component spectra in the stack collected on FIB section 1B. However, this is only true for that stack and because the data is analyzed collectively, there could be other species present in other stacks, but not in stack 1B, or subtle

differences in oxygen spectra could be present. To explore this possibility, the analysis was also carried out using three components. A full examination of the possibilities of this three-component fit is not presented here, but instead this is left as one avenue of potential interest for future work.

A major difficulty in the three-component NMF analysis is that the resulting *W* and *H* matrices are not reproducible with different (random) starting conditions for those matrices. These matrices are stable upon iteration, but not upon restarting the calculation with new initial values. Shrinking the energy range solves this problem somewhat, with the most common result shown in Fig. S2, but other results are still sometimes obtained. This is most likely due to the defocusing issue at higher energies mentioned in the main text. The other three panels of Fig. S2 show scatterplots of the weights of each component

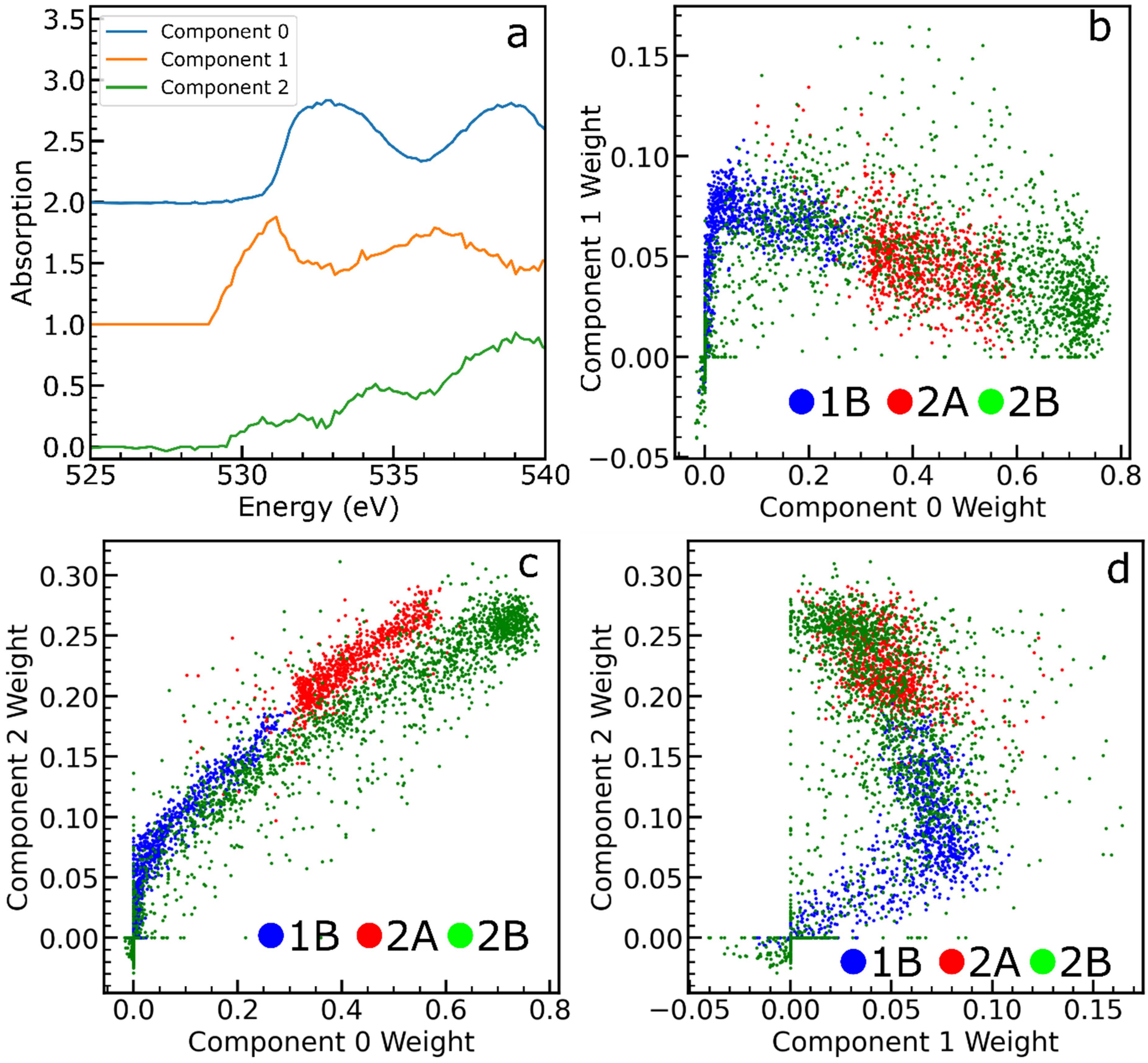

Figure S2 a) Components of three-component fit. b-d) scatter plot of the component weights for each individual pixel. Pixel color corresponds to the stack each pixel was obtained from.

The scatterplots of fig S2 which involve component 1 (Fig. S2b, S2d) are similar to what a scatterplot for the two-component fit looks like. The last comparison of components, Fig. S2c is mainly linear. This is consistent with component 1 being similar to the interfacial component of the two-component fit and components 0 and 2 adding to be similar to the bulk component. However, S2c is not completely linear, with the pixels from stacks 1B and 2A following the same trend and the pixels from stack 2B falling a bit outside of that trend. It is outside the scope of this manuscript to delve too deeply into the differences outlined here, but this dataset remains a promising source of future study.

S2. NMF Fit examples

Reproduced in Fig. S3 are examples of the NMF "fit" produced by the analysis of the O K-edge spectra of FIB section 1B. Two nearby pixels are selected, one with low interfacial content (A) and one with relatively higher interfacial content (B), each with the actual data presented as black points and the sum of the component spectra presented as a solid colored line. These "fits" reproduce the data well considering the noise of the spectra, and the subtle difference in both the data and the fits between these two locations, primarily at the pre-edge shoulder near 530 eV and the pre-edge peaks at 532 and 534 eV, show that the two-component model adequately reproduces the variations in the data. Examples for the NMF fit in the stack collected on sample 2A (Fig. S4) shows that this efficacy in reproducing variations in the data is consistent across samples.

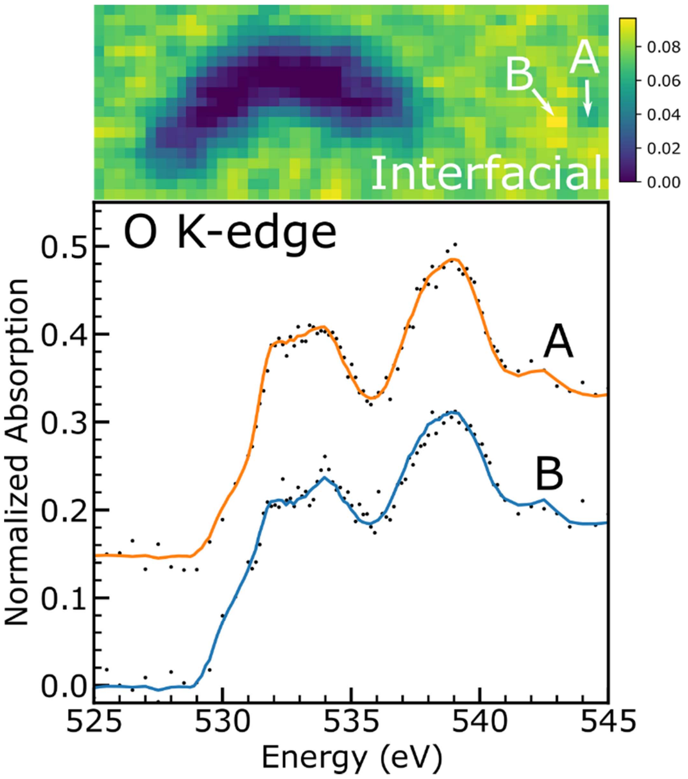

Figure S3 Top: Reproduction of the interfacial species map of FIB section 1B (Fig. 4). Positions are indicated showing the positions of the spectra shown at bottom. Bottom: data (black dots) and NMF "fits" to these spectra produced by the NMF model for O K-edge spectra. Pixel size in the image

shown is 50 nm. Fit components for spectrum A are 0.059 for the interfacial component and 0.151 for the bulk component. Fit components for spectrum B are 0.095 for the interfacial component and 0.109 for the bulk component. Note that the component weights do not add to one because the individual pixel spectra are not normalized to unity. Spectra are offset by 0.15 for clarity.

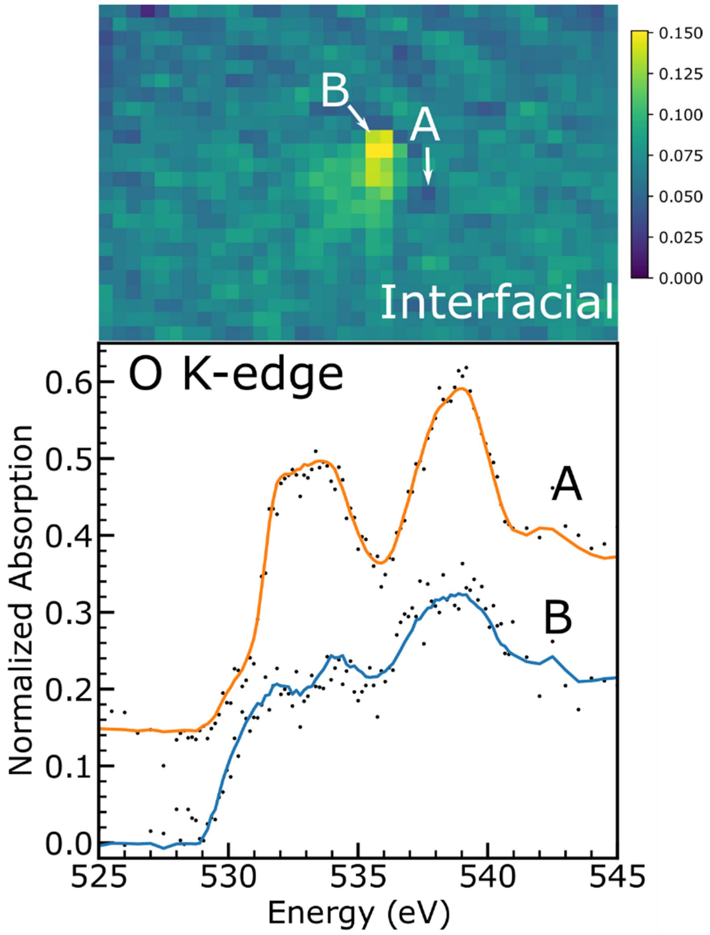

Figure S4 Top: Reproduction of the interfacial species map of FIB section 2A (Fig. 5). Positions are indicated showing the locations of the spectra shown at bottom. Bottom: data (black dots) and NMF "fits" to these spectra produced by the NMF model for O K-edge spectra. Pixel size in the image shown is 100 nm. Fit components for spectrum A are 0.040 for the interfacial component and 0.227 for the bulk component. Fit components for spectrum B are 0.149 for the interfacial component and 0.074 for the bulk component. Note that the component weights do not add to one because the individual pixel spectra are not normalized to unity. Spectra are offset by 0.15 for clarity.

S3. Barium Inclusion

Fig. S5 shows a normal contrast (top) image at the U N-edge and a Ba map generated by subtracting two images from this stack, one at the Ba M_5 peak, and one at a slightly lower energy just below the Ba M_5 peak. It is not possible to quantify the amount of Ba in this inclusion due to the lack of standards at the Ba M_5 edge to compare to, so analysis is restricted to qualitative measures here. The

oxygen K-edge at this Ba inclusion (Fig. S6) shows no difference between the area of the barium inclusion and the other portion of the sample if no vertical offset were included in the plot. This is likely because even in the barium inclusion, the FIB section is still predominantly UO_2 and so the oxygen spectrum is dominated by the bulk species.

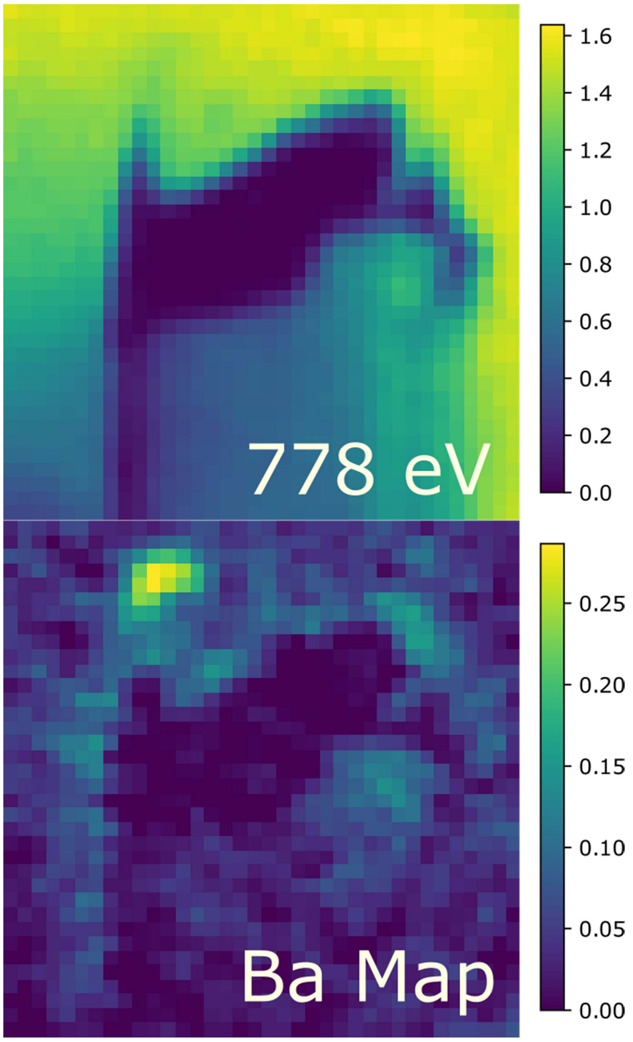

Figure S5 Top: Normal contrast image of FIB section 2B. Bottom: Ba Map generated by subtracting the image at 784.5 eV (just below the Ba M_5 peak) from the image at 785.5 eV (at the Ba M_5 peak). Pixel size in the image shown is 100 nm.

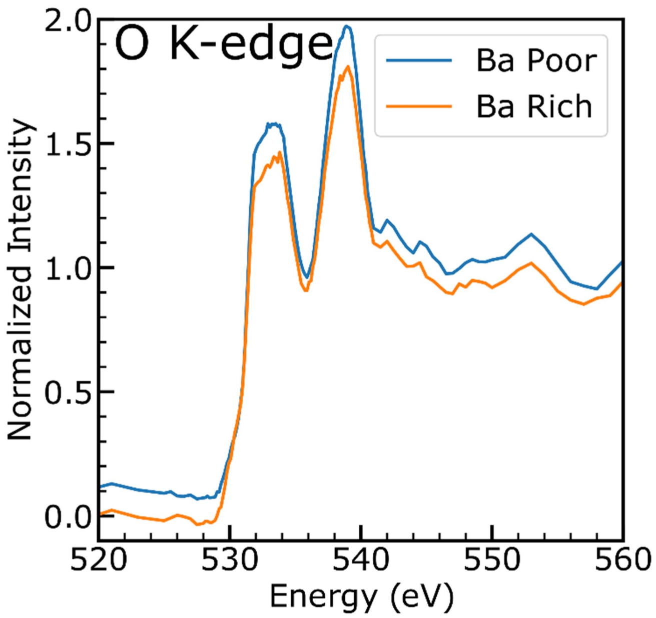

Figure S6 Comparison of O K-edge spectra in the Ba rich area (blue) of the stack collected on sample 2B to the Ba poor area (orange). The spectra are offset for clarity by 0.1. No measurable difference is distinguishable between the two samples.

The 3D distribution of sections prepared for Atom Probe Tomography is shown in Fig S7. These are conical sections prepared by FIB sectioning of regions of the fuel pellet suitable for APT. The Ce (red) is evenly distributed in each specimen.

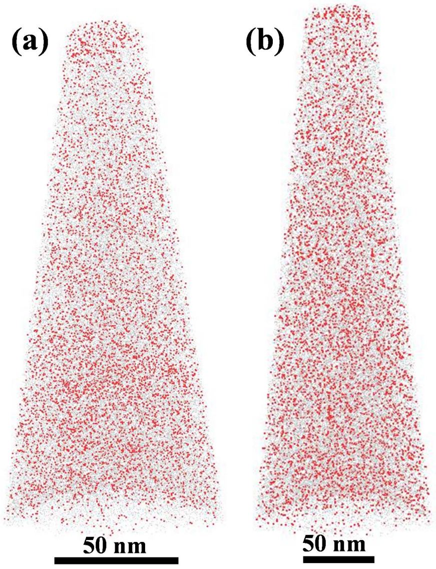

Figure S7 3D distribution of Ce (in red) ions from specimen prepared from (a) center and (b) rim location of fuel.

The data from Fig. 1 is only shown between 528 and 545 eV to emphasize the isosbestic points central to that discussion, but it is also important to show the full range of these spectra given that they are

used to normalize other spectra which must be cut off in energy before they can be normalized. This is shown in Fig. S8.

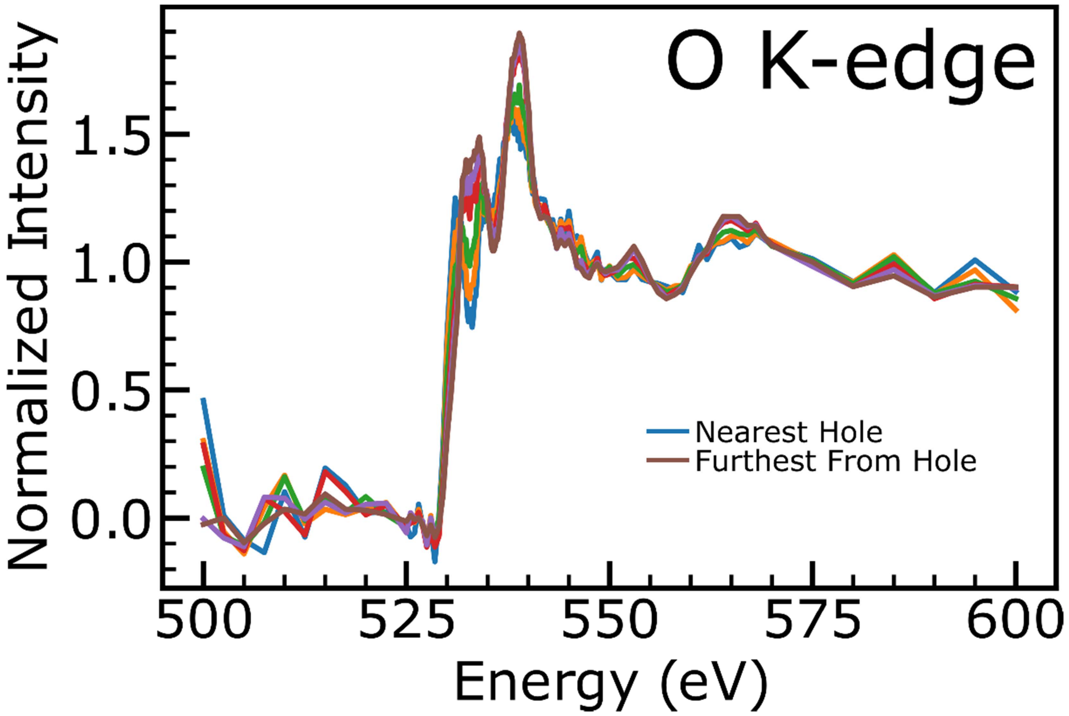

Figure S8 O K-edge spectra from Fig. 1 reproduced to show the entire range of data. The data are normalized to an edge-step of 1.
